# Patient Satisfaction After the Cleft-Lift Procedure

**DOI:** 10.7759/cureus.17686

**Published:** 2021-09-03

**Authors:** Steven C Immerman

**Affiliations:** 1 Surgery, Evergreen Surgical, Eau Claire, USA

**Keywords:** pilonidal disease, pilonidal cyst, cleft lift procedure, bascom cleft lift, pilonidal sinus, pilonidal, pilonidal surgery, pilonidal sinus surgery, pilonidal cyst surgery, cleft lift surgery

## Abstract

Introduction

Although Pilonidal disease is rarely life-threatening, it is a painful and potentially embarrassing condition that if left untreated or treated poorly, can disrupt a patient’s ability to enjoy life, function at work, develop relationships, or attend school or the military. There are several different approaches to this problem which include non-surgical measures, minimally invasive surgery, excisional surgery, or flap surgery. This article relates the experience with a surgical practice that offers only the cleft lift procedure and describes the degree of patient satisfaction with the operation.

Materials and Methods

Seven hundred and fifty-one patients were treated between 2011 and 2021. Surveys were sent out to these patients by email after at least eight weeks had elapsed from surgery. The study was closed once 500 responses were obtained. Statistical analysis was performed to determine if patients who had undergone previous pilonidal surgery (salvage group) had different opinions than the patients who did not (primary group).

Results

Of the 500 respondents, 494 (98.8%) were “extremely satisfied” or “satisfied” with their procedure; 444 (88.8%) felt that the recovery process was “very easy” or “easy” and only 56 (11.2%) felt that it was “difficult but worth it” or “really hard”. Four hundred and one (80.2%) felt that the activity restrictions were “minimal, I was back to normal activity very quickly”; 438 (89.4%) felt that the scar looked “really good” or answered, “it’s fine, not an issue for me. I’m just glad to be done with this”. Whether the patients had previous failed surgery or not, the vast majority (78.2% and 79.6% respectively) felt that the cleft lift was an appropriate first operation for pilonidal disease; and statistical analysis failed to show any significant differences in opinions between the primary and salvage groups on any of the questions. The few patients who ultimately were dissatisfied with the procedure were unhappy with the cosmetic appearance of the scar and shape of the buttocks. By comparing the demographic characteristics of the respondents to the entire cohort, we found them to be similar groups, suggesting that the respondents were representative of the group as a whole.

Conclusion

A correctly performed cleft lift operation provides a solution that is very well accepted by patients, specifically in regard to recovery time, appearance, appropriateness, and overall satisfaction.

## Introduction

In our pilonidal clinic, we strive to perform a cosmetically acceptable procedure that removes all active disease, prevents recurrence, and provides a solution with which the patient is satisfied. If the patient is not pleased with the cure, then the procedure was not a success for that individual. This paper is a report on patient satisfaction with the cleft lift operation over a 10-year period and includes survey responses from 500 patients.

The cleft lift procedure was described by Dr. John Bascom in 1987. This was a modification of the Karydakis procedure, which is an off-midline closure operation, described by Dr. George Karydakis in 1973. The cleft lift operation has been found to be effective in treating pilonidal disease as both a primary procedure and as a salvage operation in patients who have failed other operations [1]. The key technical points of the operation are that the active pilonidal disease is removed, the entire cleft is flattened, and the incision is moved off the midline.

## Materials and methods

With the advent of internet-based surveying techniques and the prevalence of email communication the ability to get patient’s opinions on their surgery and recovery has been enhanced. We began this survey process in 2011 using a Survey Monkey survey which was sent to all patients approximately two to three months after their operation. Surveying patients within this time frame would allow their recovery issues to be fresh in their memories, but not so soon that primary healing had not occurred. This is not a study of complications or recurrences, but rather the patient’s subjective opinion regarding the procedure. The wording of the questions and proposed answers was specifically structured to use only a small number of questions in non-clinical language, and include “other” fields for patients to elaborate on their answers and mention issues that the questions might not address. The patients in this cohort consisted of patients who chose the cleft lift as their first excisional procedure after diagnosis with pilonidal disease (primary group); and also patients who had one or more previous excisional procedures including minimally invasive procedures, open and closed excisions, and flap procedures (salvage group). Incision and drainage operations for pilonidal abscesses were not considered excisional procedures.

All of the patients in this cohort had the cleft lift operation done by the author. The key factors in performing this procedure are to flatten the entire gluteal cleft, remove all active pilonidal disease, and position the final incision well off the midline [2-3].

The survey consisted of fourteen questions, nine of which were non-clinical and consisted of: how they found our clinic, how easily they were able to have surgery scheduled, and what they thought of our follow-up process, and some demographic questions. The five clinical questions, which are the topic of this paper, are related to the patient’s satisfaction with how they felt about: the procedure overall, the recovery process, the post-op activity restrictions, the appearance of the scar, and the appropriateness of the cleft lift as the first operation for a patient with pilonidal disease. The question about appropriateness was posed differently to the primary vs. salvage groups because we felt that patients who had previously failed surgery (salvage group) might have a different viewpoint than those for whom this was their first excisional operation (primary group). All questions were analyzed looking for differences between these two groups. The survey monkey data was entered onto a Microsoft Excel spreadsheet and chi-square tests were used to calculate p-values and statistical significance.

The survey included one open-ended question asking for opinions about the care they received at our clinic and for “Any advice for us to serve you better, or advice for other patients?”. Narrative answers were reviewed and categorized as appropriate for the question being posed.

## Results

We ended the study period once 500 surveys were returned. During the time frame of the study, this included 751 patients, of whom 500 (66.6%) responded. Not all patients completed all questions, but each of the five questions for this study had at least 490 answers. Sixty-eight patients were not comfortable providing their name along with the survey, even though the majority of the responses from these 68 patients were quite positive about their experience.

Overall patient satisfaction with the procedure

**Figure 1:**
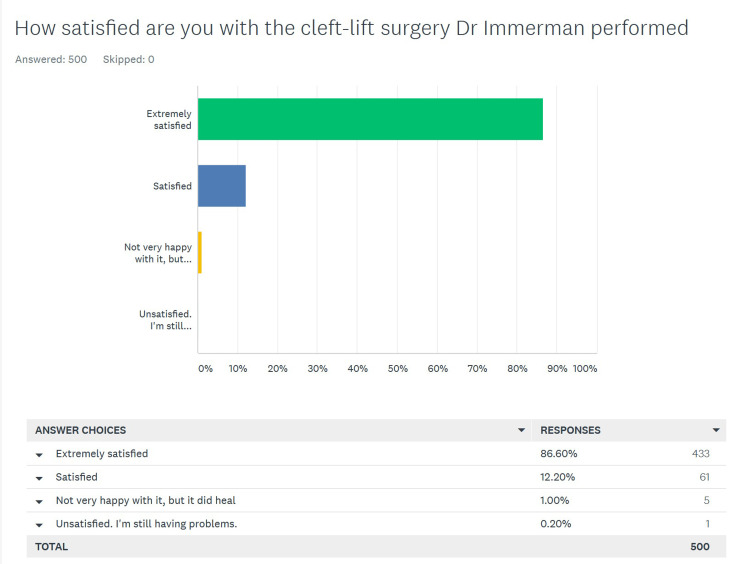
Overall patient satisfaction with the cleft lift operation

There were 500 responses to this question. The wording of the question and choices is demonstrated in Figure 1, and the breakdown of the data between the primary and salvage groups is shown in Table 1.

**Table 1:** Overall satisfaction with the cleft lift operation showing differences between primary and salvage patients

	All Patients		Primary		Salvage	
Total number of patients	500		223		277	
Extremely satisfied	433	86.60%	186	83.41%	247	89.17%
Satisfied	61	12.20%	32	14.35%	29	10.47%
Not very happy with it, but did heal	5	1.00%	5	2.24%	0	0
Unsatisfied. I'm still having problems	1	0.20%	0	0.00%	1	0.4%
Total of Extremely satisfied and Satisfied	494	98.80%	218	97.76%	276	99.64%

We looked at the responses to this question as either positive or negative. The positive responses were “extremely satisfied” or “satisfied”, and 494 (98.8%) of the patients chose one of these. Only six (1.2%) answered that they were “not very happy with it, but it did heal” or “unsatisfied, I’m still having problems”.

When divided into primary vs. salvage patients, 276 out of 277 (99.6%) of the primary patients gave a positive response, vs. 218 out of 223 (97.6%) of the salvage patients, the difference was not statistically significant (p = 0.09).

Patients’ opinion of the difficulty with recovery

**Figure 2:**
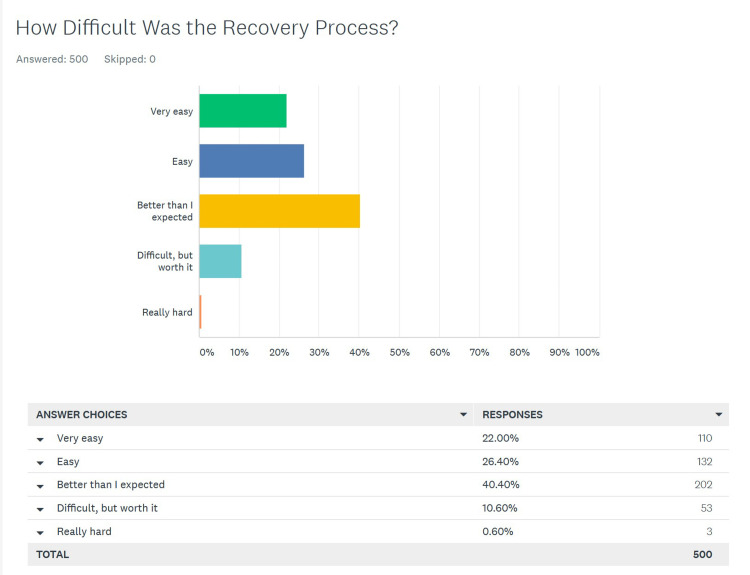
Patients' opinion regarding the difficulty of the recovery process after a cleft lift

There were also 500 responses to this question and the wording and graphical view of the data is seen in Figure 2.

For this question, we felt that the answers indicating that the recovery was “very easy”, “easy”, and “better than I expected” were all positive responses, and that “difficult but worth it” and “really hard” were considered negative (Table 2).

**Table 2:** Patients' opinion of the recovery process after a cleft lift and differences between primary and salvage groups

	All Patients		Primary		Salvage	
Total number of patients	500		223		277	
Very easy	110	22.00%	36	16.14%	74	26.71%
Easy	132	26.40%	55	24.66%	77	27.80%
Better than I expected	202	40.40%	108	48.43%	94	33.94%
Difficult, but worth it	53	10.60%	22	9.87%	31	11.19%
Really hard	3	0.60%	2	0.90%	1	0.36%
Total of Very easy, Easy, Better than I expected	444	88.80%	199	89.24%	245	88.45%

Overall, 444 (88.8%) respondents gave a positive response, and 56 (11.2%) gave a negative response. The positive response rate for primary vs. salvage patients were rates of 89.2% and 88.5% respectively, which were not statistically different (p > 0.05). There was the option to add comments to this question and 170 patients had more to say. We specifically searched these comments for any indication of chronic pain; only two patients said they had minor discomfort with sitting at two months post-op.

Patients’ opinion of activity restrictions after surgery

**Figure 3:**
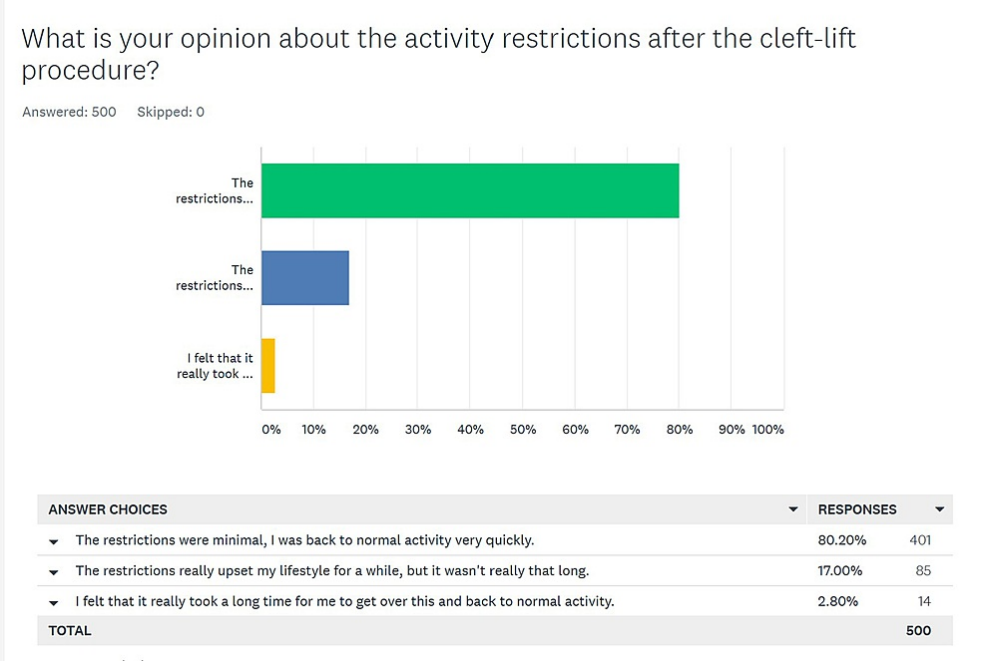
Patients' opinion about post-op activity restrictions after a cleft lift

We attempted to minimize the restrictions placed on patients during the postoperative period. The main restrictions were to avoid contact sports, jogging, and biking for six weeks. Sitting, showering, and normal ambulatory activity was encouraged immediately after surgery. The survey responses can be seen in Figure 3, and again there were 500 responses. We considered the responses “the restrictions were minimal, I was back to normal activity very quickly” and “the restrictions really upset my lifestyle for a while, but it wasn’t really that long” as positive responses, and 486 (97.2%) of patients chose these two options. Only 14 (2.8%) answered “I felt that it really took a long time for me to get over this and back to normal activity”. The breakdown of answers from primary and salvage patients can be seen in Table 3, and there was essentially no difference (p = 0.54) between the two groups.

**Table 3:** Patients' opinion of post-op activity restrictions after a cleft lift and differences between primary and salvage groups

	All Patients		Primary		Salvage	
Total number of patients	500		223		277	
The restrictions were minimal, I was back to normal activity very quickly.	401	80.20%	175	78.48%	226	81.59%
The restrictions really upset my lifestyle for a while, but it wasn't really that long.	85	17.00%	40	17.94%	45	16.25%
I felt that it really took a long time for me to get over this and back to normal activity.	14	2.80%	8	3.59%	6	2.17%
Total "minimal" or "wasn't really that long"	486	97.20%	215	96.41%	271	97.83%

Patients' opinions regarding the appropriateness of the cleft lift as a patient’s first operation for pilonidal disease

One of the most controversial topics regarding the cleft lift is its place within the treatment algorithm for pilonidal disease. At our clinic we recommend this for any patient with pilonidal disease who understands the operation, the post-operative restrictions, and the cosmetic consequences; we make sure our patients are well educated regarding the procedure and refer them to a comprehensive website created by the author which includes many post-operative photos [4]. We posed this question differently to the primary vs salvage groups.

Primary Patients

**Figure 4:**
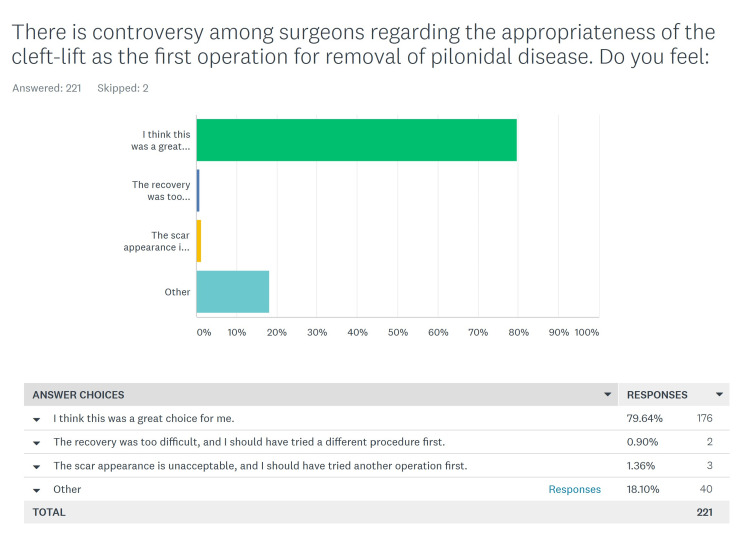
Opinion of Primary group of patients regarding the appropriateness of the cleft lift as a first operation for pilonidal disease

These were the 221 patients who had not had previous excisional surgery. The exact question posed and possible answers are shown in Figure 4. We had an “other” choice for patients to write in their answers, and although most of these narratives closely corresponded to the first three answers, patients often wanted to expand on their feelings. These “other” answers were analyzed and added to one of the first three categories based on the content of the answer. We found that 210 (95%) of patients had a positive response that corresponded to “I think this was a good choice for me”; three (1.4%) felt “the recovery was too difficult”, and three (1.4%) felt they “should have tried another operation first, the scar is unacceptable” (Table 4). Five patients gave ambiguous narrative answers and were not included in the totals. When the details of the three patients who were unhappy with the scar were reviewed, we found that there were two males and one female (which corresponds to our overall male vs. female ratio), they had no wound healing problems, and photos showed that the contour and the scar was actually optimal for this procedure.

**Table 4:** Primary patients' opinions regarding the cleft lift as a patient's first operation for pilonidal disease, including "other" answers

Total number of patients answered	221	
I think this was a great choice for me.	210	95.0%
The recovery was too difficult, and I should have tried a different procedure first.	3	1.4%
The scar appearance is unacceptable, and I should have tried another operation first.	3	1.4%

Salvage Patients

**Figure 5:**
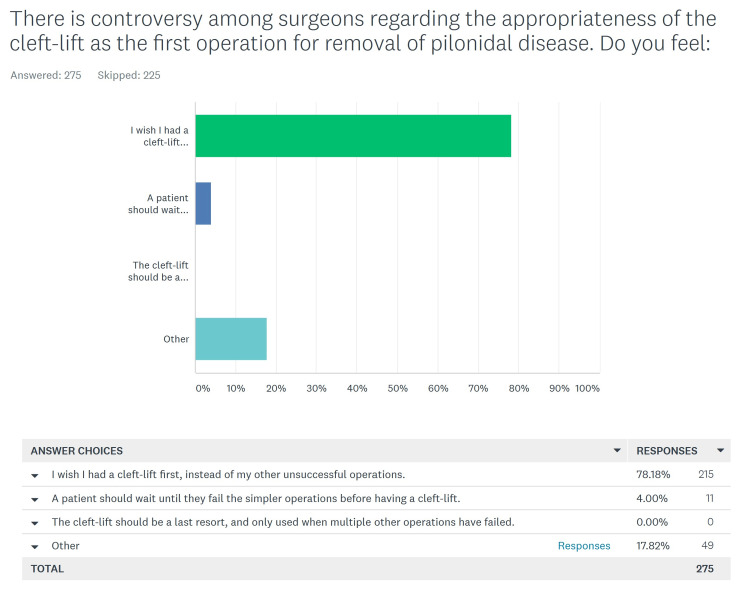
Salvage patients' opinion of the cleft lift as the primary procedure for pilonidal disease

There were 275 patients who had one or more previous operations before the cleft lift at our clinic. The question for these patients is seen in Figure 5, and again there was an “other” category that was reviewed and categorized. Many of these (37) were quite enthusiastic about the cleft lift, but eight gave thoughtful answers indicating that they thought a patient should try other procedures. Four were non-committal and excluded; none felt it should be a last resort. We found that 252 (91.6%) of the patients selected the answer “I wish I had a cleft-lift first, instead of my other unsuccessful operations”; while 19 (6.9%) felt that “A patient should wait until they fail the simpler operations before having a cleft-lift”. No patients selected the answer “The cleft-lift should be a last resort, and only used when multiple other operations have failed” (Table 5). Several of the narrative answers expressed incredulity that there was controversy about this, such as this answer:

Why is there controversy? I wasted $5,000 on surgery, gauze, gasoline, and eight months of my life taking showers twice a day, getting stung by silver-nitrate weekly, and also not sitting properly, only to have a second surgery all over again. The cleft-lift should be standard practice, especially considering it's almost 100% success rate.

**Table 5:** Salvage patient's opinion of the cleft lift as the primary operation, including "other" responses

Total number of patients answered	275	
I wish I had a cleft-lift first, instead of my other unsuccessful operations.	252	91.6%
A patient should wait until they fail the simpler operations before having a cleft-lift.	19	6.9 %
The cleft-lift should be a last resort, and only used when multiple other operations have failed	0	0.0%

Patient’s opinion of the appearance of the buttocks and scar

**Figure 6:**
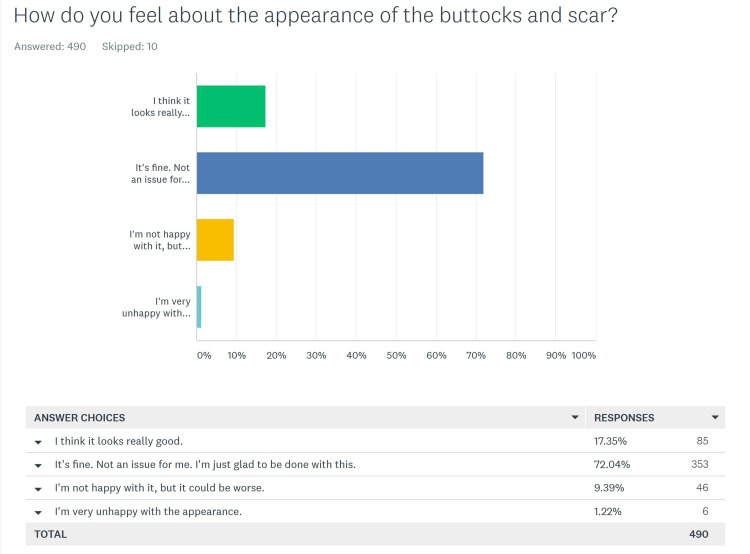
Patients' opinion of the buttocks and scar appearance

Only 490 patients answered this question (Figure 6). The overall opinion of patients regarding the appearance was that 85 (17.3%) felt that it “looks really good”, 323 (72%) felt that “it’s fine. Not an issue for me. I’m just glad to be done with this”. Forty-six (9.4%) selected “I’m not happy with it, but it could be worse”, and six (1.2%) were “very unhappy with the appearance”. Of these last six, only one of the patients overlapped with the three in the primary group who indicated that “the scar appearance was unacceptable”, so there were five others who felt that way; three males and two females. But, in spite of their negative feelings about the scar, these five rated their overall satisfaction with the procedure as “extremely satisfied” (four) and “satisfied” (one). Again, there was no statistical difference between the answers from the salvage vs. primary groups (p = 0.75).

Open-ended narrative question about their experience

When asked about “any advice for us to serve you better, or advice for other patients?”, 339 patients chose to elaborate on their answers to this survey. These answers were categorized as positive, negative, or neutral. Neutral comments were either observations without praise or criticism, or combined the two, such as “The first few days were very painful, but after that everything went well and I’m very pleased”. There were 322 (95%) positive, 12 (4%) neutral, and one (0.3%) negative. The single negative comment had to do with the amount of preoperative information given. Notably, none of these comments indicated that patients were experiencing chronic pain at the site of the operation.

## Discussion

There is much controversy about the optimal procedure for treating pilonidal disease [5] because the results and recovery issues vary significantly between procedures and between surgeons [6]. This paper was not meant to supply a definitive answer to that controversy, but rather to describe the satisfaction level of patients after having the cleft lift procedure when performed in a high-volume pilonidal clinic; and what aspects of the procedure might cause dissatisfaction. This information can be used as additional data when selecting the right operation for an individual patient. In this group of patients, the cleft lift was the only pilonidal operation performed during this ten-year period except for incision and drainage of acute abscesses. Some may view this approach as unusual, but it does give a unique view of the success of this procedure. We recently published a paper describing clinical parameters of success and failure with the cleft lift in a large group of patients [7], and in this paper, the overall success rate was 96.6%; but just because the surgeon views the operation as success does not mean the patient is in agreement. This paper helps to answer that question, with 98.8% of patients providing a response indicating satisfaction with the procedure.

As surgeons, most of the operations we perform have high success rates and the majority of patients are satisfied with the results. However, with pilonidal disease many of the operations require prolonged wound care, and recurrence rates have been reported up to 67.9% at 20 years [6]. This leads to many unsatisfied patients, and corresponding stress for surgeons, office staff, and patient’s families. This degree of stress and dissatisfaction can easily dissuade surgeons from agreeing to treat pilonidal patients, and often surgery is delayed or not recommended at all. This survey demonstrates that a very high satisfaction rate can be obtained with the right operation done by an experienced surgeon.

For the few patients who were ultimately unhappy with the cleft lift procedure, it was based on their experience regarding the change in the shape of the buttocks and the scar, and this is very understandable. However, it does not mean that they would have been happier with the results from any of the other operations for pilonidal disease, and more studies of patient satisfaction from other operations are needed to put this in context. Nonetheless, the vast majority of these patients gave either positive answers regarding the appearance; or had a mature outlook on the situation and indicated that it wasn't a significant issue for them, and they were just glad to be done with their battle with pilonidal disease. The few that were unhappy with the appearance had good results - but their subjective opinion of the appearance was negative; further supporting the concept that there can be a disparity between the surgeon’s and the patient’s idea of success, and further highlighting the importance of preoperative discussion.

The most controversial questions in this study seek the patient’s opinion on whether, in retrospect, they agree that they chose the right operation for their problem and whether they feel that the cleft lift is a reasonable choice as a patient’s first operation for this disease. In our clinic, we take an extremely positive view regarding the broad application of the cleft lift procedure. If our surveyed patients disagreed with this concept, this would have required re-evaluation. If we combine the responses from both the primary and salvage groups for this question, 496 (93.8%) feel that this is a reasonable first choice for a patient with pilonidal disease. It was notable that although 7.2% of the Salvage patients felt that a patient should fail a simpler operation before having a cleft lift, none of the patients felt that “The cleft-lift should be a last resort, and only used when multiple other operations have failed”.

There have been other studies of patient satisfaction after pilonidal operations. Gaiser et al., correlated satisfaction after wide excision to gender, pain killer intake, and smoking cessation, and reported a mean healing time of 20 months [8]. Doll et al. reported on 583 German military patients and the relationship between wound healing time and recurrence on patient satisfaction [9]. These patients had open or closed excision or marsupialization, were followed for up to 20 years after surgery, and overall showed satisfaction of 8.2 on a one to 10 scale. They found that satisfaction ultimately had to do with non-recurrence more than difficult or prolonged recovery. Mueller et al. presented a study analyzing seventy patients’ satisfaction after the Limberg Flap procedure and found a mean overall satisfaction of 7.8 on a one to 10 scale [10]. They also found that cosmesis was a significant factor in patient dissatisfaction. It is difficult to compare results from these studies because of varying the time frames of the surveys, and different question formats.

A limitation of this study was the response rate of our patients, which was 66.6%. However, the demographics of the respondents matched the cohort as a whole, so we feel that this is a representative sample of patients. The lack of a universally accepted pilonidal classification system and a validated tool for assessing patient outcomes [11] hampers the ability to compare various studies of outcome and patient satisfaction.

## Conclusions

When performed in a high-volume pilonidal clinic, patients indicate a high satisfaction rate with the cleft lift procedure. The few instances of dissatisfaction are related to the patients' opinion of the buttocks and scar. Overall, patients feel that the recovery from surgery and the activity restrictions are quite manageable. They feel that it is an appropriate option as a primary procedure. The high satisfaction with the procedure, recovery, and appearance, was the same whether or not they had previously failed surgery.

## References

[1] Bascom J, Bascom T (2007). Utility of the cleft lift procedure in refractory pilonidal disease. Am J Surg.

[2] Bascom J, Bascom T (2002). Failed pilonidal surgery: new paradigm and new operation leading to cures. Arch Surg.

[3] Kraft CT, Khansa I, Janis JE (2020). Practical management of pilonidal disease. Plast Reconstr Surg Glob Open.

[4] (2021). The cleft lift procedure. https://pilonidal.net/pilonidal-disease-cleft-lift-surgery/.

[5] Vartanian E, Gould DJ, Lee SW, Patel KM (2018). Pilonidal disease: classic and contemporary concepts for surgical management. Ann Plast Surg.

[6] Stauffer VK, Luedi MM, Kauf P (2018). Common surgical procedures in pilonidal sinus disease: a meta-analysis, merged data analysis, and comprehensive study on recurrence. Sci Rep.

[7] Immerman SC (2021). The bascom cleft lift as a solution for all presentations of pilonidal disease. Cureus.

[8] Gaiser MR, Lee SB, Enk A, Schrott P, Weisser H (2013). Surgical intervention of pilonidal sinus: impact on patients' postoperative satisfaction and return to work time. Eur J Dermatol.

[9] Doll D, Luedi MM, Evers T, Kauf P, Matevossian E (2015). Recurrence-free survival, but not surgical therapy per se, determines 583 patients' long-term satisfaction following primary pilonidal sinus surgery. Int J Colorectal Dis.

[10] Müller K, Marti L, Tarantino I, Jayne DG, Wolff K, Hetzer FH (2011). Prospective analysis of cosmesis, morbidity, and patient satisfaction following Limberg flap for the treatment of sacrococcygeal pilonidal sinus. Dis Colon Rectum.

[11] Tabone RA, Wysocki AP (2019). Patient reported outcomes in pilonidal disease- results of a patient survey. Pilonidal Sinus Journal.

